# Enhanced Recovery After Surgery (ERAS) Pathways in Elective Total Joint Arthroplasty

**DOI:** 10.7759/cureus.91481

**Published:** 2025-09-02

**Authors:** Zabrang Ishaku, Daniel I Koshy, Munir Adamu Bala

**Affiliations:** 1 Trauma and Orthopaedics, Barts Health NHS Trust, London, GBR; 2 Trauma and Orthopaedics, Queen Mary University of London, London, GBR; 3 Trauma and Orthopaedics, The Royal London Hospital, London, GBR; 4 General Surgery, Norfolk and Norwich University Hospital, Norwich, GBR

**Keywords:** eras protocols, orthopedic rehabilitation, primary total knee arthroplasty, total hip arthroplasty (tha), total hip replacement (thr)

## Abstract

Enhanced recovery after surgery (ERAS) protocols are increasingly used in total hip and knee arthroplasty to improve outcomes, reduce complications, and shorten hospital stays. This involves a multidisciplinary, evidence-based approach covering the preoperative to postoperative period. This review explores the current literature on ERAS implementation in elective total hip arthroplasty (THA) and total knee arthroplasty (TKA), focusing on clinical outcomes such as length of stay (LOS), opioid use, complications/readmissions, and other key components such as anesthetic standardization, use of opioid-sparing analgesia agents, early mobilization, and patient satisfaction.

We performed a literature search of PubMed, Embase, and Google Scholar databases (last searched May 2025) for studies evaluating the use of ERAS protocols in elective THA and TKA. Relevant articles, including clinical trials, observational studies, case series, case reports, and review articles, were identified without geographic restrictions, limited to English-language publications from 2000 to 2025. Key search terms included “enhanced recovery after surgery,” “ERAS,” “fast-track,” “rapid recovery,” “total joint arthroplasty,” “total knee replacement,” “total hip replacement,” and “outcomes.” The selection of articles was based on relevance to the topic, emphasizing clinical outcomes. No formal quantitative synthesis was performed, given the narrative scope.

The included literature (spanning approximately 294,000 patients across multiple studies) consistently demonstrates that ERAS pathways significantly reduce LOS by one to three days without increasing readmission or complication rates. Multimodal, opioid-sparing analgesia regimens lead to superior pain control and reduced opioid exposure, enhancing early mobilization and minimizing adverse effects. Functional recovery is accelerated, with many ERAS patients ambulating within hours of surgery. Patient-reported outcomes and satisfaction are high, and early quality-of-life improvements are commonly observed. ERAS implementation results in substantial cost savings from a systems perspective, largely due to shorter inpatient stays and fewer postoperative complications. The evolution of ERAS has also facilitated the growth of outpatient (same-day discharge) arthroplasty in appropriately selected patients.

ERAS in total joint arthroplasty (TJA) represents a paradigm shift in perioperative care, combining safety, efficiency, and patient-centred recovery. Its implementation leads to improved clinical outcomes and enhanced healthcare value, supporting its continued expansion and adoption as a global standard of care in hip and knee arthroplasty.

## Introduction and background

Total joint arthroplasty (TJA) - primarily total hip and knee replacements - is among the most common and cost-intensive elective surgeries worldwide. Historically, recovery from hip or knee replacement involved prolonged bed rest, in-hospital rehabilitation, and a length of stay (LOS) often exceeding a week. Over the past two decades, there has been a paradigm shift toward “fast-track” or enhanced recovery after surgery (ERAS) protocols in arthroplasty, aiming to improve patient outcomes and efficiency. ERAS is a multimodal, evidence-based perioperative care pathway designed to mitigate the stress of surgery and hasten recovery [1]. Key ERAS elements in TJA include preoperative patient education and medical optimization (e.g. managing anemia, glucose, nutrition), minimal fasting with carbohydrate loading, use of regional anesthesia and opioid-sparing multimodal analgesia, prophylaxis against nausea and venous thromboembolism (VTE), surgical techniques that reduce tissue trauma, early removal of drains/catheters, early oral intake, and early mobilization on the day of surgery (Table 1). By coordinating these interventions, ERAS pathways help patients regain function faster and with fewer complications than traditional protocols.

**Table 1:** Key elements of ERAS protocols in total joint arthroplasty (TJA) and their benefits. LOS: length of stay; TKA: total knee arthroplasty; THA: total hip arthroplasty; PONV: postoperative nausea and vomiting; POD1: postoperative day 1; DVT: deep vein thrombosis. Source: Created using data from references [2-9].

ERAS Component	Implementation in TJA	Outcome/Benefit
Preoperative education and optimization	Patient counseling, home-based exercise “prehab” 4-8 weeks before surgery, nutrition, and smoking cessation plans	Reduces anxiety and improves pre-surgical fitness. Meta-analysis shows prehab can modestly improve pre-op pain/function and significantly reduce LOS (by ~0.4 days in TKA) [2], though long-term functional benefits are unclear.
Regional anesthesia and blocks	Spinal anesthesia instead of general when feasible; addition of peripheral nerve blocks (e.g., adductor canal block for TKA, peri-capsular nerve group block for THA)	Lowers early postoperative pain and nausea compared to general anesthesia [3]. Preserves motor function for quicker mobilization [4]. Either spinal or carefully managed general anesthesia can be used for same-day discharge with comparable safety [3].
Multimodal analgesia (opioid-sparing)	Scheduled non-opioid analgesics (acetaminophen, NSAIDs/COX-2 inhibitors), gabapentinoids, local anesthetic infiltration, ± corticosteroids; minimal IV opioids	Provides effective pain control while dramatically reducing opioid requirements. ERAS studies report significantly lower postoperative opioid consumption (often >50% reduction in morphine equivalents) with equal or better pain relief [3]. This leads to fewer opioid-related side effects (sedation, nausea) and supports early rehab.
Tranexamic acid (TXA)	IV TXA 10-20 mg/kg before wound closure (± additional dose or topical TXA); some protocols continue TXA doses for 1-3 postoperative days	Dramatically reduces perioperative blood loss and transfusion rates. TXA has not increased thromboembolism risk in large analyses and may improve the early range of motion by limiting hemarthrosis [5]. TXA is now standard in ERAS for TJA, barring contraindications.
Minimally invasive surgical techniques	Meticulous hemostasis; avoid routine drains; limited tourniquet use (or release before closure) in TKA; tissue-sparing approaches when possible	Less postoperative pain and swelling, facilitating faster mobilization. Omitting suction drains can reduce anemia and doesn’t increase wound complications in modern practice [6]. No particular prosthesis type or approach has proven superior for ERAS, so the focus is on surgical efficiency and avoiding unnecessary tissue trauma.
Active prevention of PONV and ileus	Multimodal prophylaxis for nausea (e.g., dexamethasone, ondansetron); early oral intake on the day of surgery as tolerated	Reduces nausea and vomiting, which are common barriers to early mobilization and discharge. Aggressive PONV prophylaxis is a recommended ERAS element [7]. Early feeding (or at least clear fluids) helps maintain gut function and hydration, contributing to shorter hospital stays.
Early mobilization	Physical therapy beginning on the day of surgery (within 4-6 hours if possible); weight-bearing as tolerated with a walker; daily rehab goals	Accelerates functional recovery - most ERAS patients ambulate on surgery day or POD1. Early PT leads to shorter LOS (often by 1-2 days) and better in-hospital mobility (longer walking distance, quicker Timed Up-and-Go) [8]. Also lowers risk of complications like pneumonia, DVT, and muscle deconditioning.
Discharge planning and follow-up	Criteria-based discharge (e.g., independently mobile with assistive device, pain controlled on oral meds); caregiver education; phone follow-up 24-72 hours after discharge	Ensures safe transition home and high patient satisfaction. ERAS patients overwhelmingly prefer the accelerated recovery process and would choose it again in studies (90%+ satisfaction rates reported) [9]. Early follow-up helps promptly address any issues, which contributes to readmission rates comparable to traditional care.

Conventional perioperative care - characterized by extensive opioid use, delayed mobilization, and a siloed approach to recovery - often prolonged LOS and risked excess morbidity (e.g., sedation, ileus, hospital-acquired infections). In contrast, ERAS emphasizes a proactive, patient-centered recovery process. Multidisciplinary ERAS teams (surgeons, anesthesiologists, nurses, physiotherapists, etc.) work in unison to implement best practices consistently for each patient. This coordinated care has yielded measurable improvements: modern ERAS programs for THA and TKA have consistently shown shorter hospital stays without higher readmissions, reduced pain scores and opioid requirements, earlier mobilization, and at least equivalent (if not improved) rates of complications and patient satisfaction [10,7]. International ERAS guidelines have also been developed - for example, a 2020 ERAS Society consensus identified about 17 key perioperative elements (from preoperative optimization and anesthetic technique to transfusion avoidance, opioid-sparing analgesia, and early mobilization) as best practices for hip and knee replacement [5]. 

## Review

Methodology

Literature Search Strategy

We conducted a comprehensive literature search to identify relevant publications on ERAS in total hip and knee arthroplasty. Searches were performed in PubMed/MEDLINE, Embase, and Google Scholar (last search May 30, 2025), using combinations of keywords such as “enhanced recovery after surgery,” “ERAS,” “fast-track,” “rapid recovery,” “total joint arthroplasty,” “total knee replacement,” “total hip replacement,” and “outcomes.” We also manually screened reference lists of key articles to capture any additional studies. The search was restricted to English-language articles from 2000 to 2025 (Table 2).

**Table 2:** Database search strings.

Database	Search Strategy (Keywords/Terms)	Filters Applied	Hits Retrieved
PubMed/MEDLINE	(“Enhanced Recovery After Surgery”[Mesh] OR “enhanced recovery after surgery” OR ERAS OR “fast-track” OR “rapid recovery”) AND (“Arthroplasty, Replacement, Knee”[Mesh] OR “Arthroplasty, Replacement, Hip”[Mesh] OR “total knee arthroplasty” OR “total hip arthroplasty” OR “total joint arthroplasty” OR “knee replacement” OR “hip replacement”)	English only; 2000-2025	128
Embase	(‘enhanced recovery after surgery’/exp OR ERAS OR “fast track” OR “rapid recovery”) AND (‘total hip arthroplasty’/exp OR ‘total knee arthroplasty’/exp OR “total joint arthroplasty” OR “hip replacement” OR “knee replacement”)	English only; 2000-2025	176
Google Scholar	allintitle: (“enhanced recovery after surgery” OR ERAS OR “fast-track” OR “rapid recovery”) (“total hip arthroplasty” OR “total knee arthroplasty” OR “total joint arthroplasty” OR “hip replacement” OR “knee replacement”)	English only; 2000-2025	≈4,200 (first 300 screened)

Inclusion and Exclusion Criteria

We included all study types that provided data on ERAS pathways in primary THA and TKA, focusing on comparative outcomes versus conventional care. This included randomized controlled trials (RCTs), cohort studies, meta-analyses, and large registry studies (American Joint Replacement Registry). Key outcomes of interest were hospital LOS, pain scores and opioid use, postoperative complications, readmission rates, functional recovery, patient-reported outcome measures (PROMs), and cost-effectiveness. Non-orthopedic ERAS studies and non-English studies were excluded to maintain feasibility. When multiple reports from the same institution or dataset were encountered, we included the most comprehensive report to avoid overlapping data.

Study Selection and Data Synthesis

Initial database search yielded approximately 4504 articles (128 from PubMed, 176 from Embase, and approximately 4,200 from Google Scholar). After title/abstract screening and application of inclusion criteria, approximately 40 relevant full-text articles were identified for detailed review. Ultimately, 31 studies were included in our synthesis. Study selection was conducted by two independent reviewers to reduce selection bias, with any disagreements resolved through discussion. No formal scoring of methodological quality (e.g., no risk of bias tool) was performed, given the heterogeneity of study designs; however, the strength of evidence was considered when formulating conclusions (with RCTs and meta-analyses given the greatest weight). No formal Preferred Reporting Items for Systematic Reviews and Meta-Analyses (PRISMA) flow diagram was used, given the narrative scope, but our selection process yielded a broad sample of high-quality evidence in this field. We adhered to Scale for the Assessment of Narrative Review Articles (SANRA) best-practice guidelines for narrative reviews, ensuring methodological transparency, balanced appraisal, and structured synthesis. Our synthesis is organized thematically, and we acknowledge limitations of evidence (many studies are observational, possibly subject to selection or publication bias, and limiting to English-language publications may introduce language bias, and we discuss generalizability and limitations of the included studies later in this review).

Anaesthetic and analgesic strategies

Recent studies have refined perioperative anesthetic techniques within ERAS. A multicenter retrospective analysis of 105 spinal analgesia and 105 general anaesthesia (210 patients) in the United States (U.S.) compared spinal versus general anesthesia in same-day total hip and knee arthroplasty at an ambulatory surgery center (ASC). Both approaches achieved very high same-day discharge (SDD) rates (>98% success) and similar 90-day complication rates. However, differences emerged in recovery profiles: short-acting mepivacaine spinal blocks and general anesthesia enabled faster post-anesthesia care unit (PACU) discharge than long-acting bupivacaine spinal blocks, and the spinal anesthesia patients had markedly lower pain scores and nausea in the first one to two hours after surgery. The authors concluded that either modality can be used for outpatient arthroplasty with comparable safety, but spinal anesthesia provided better early analgesia (at the cost of slightly prolonged recovery-room stay if long-acting agents were used) [3]. This data supports ERAS recommendations favoring short-acting regional anesthetics (or carefully titrated lighter general anesthesia) plus aggressive multimodal analgesia to facilitate early mobilization and discharge.

In practice, ERAS protocols now routinely employ regional nerve blocks and local anesthetic infiltration as part of a multimodal pain strategy. For example, adductor canal blocks for TKA or peri-capsular nerve group (PENG) blocks for THA provide analgesia while preserving motor function [4]. A systematic review and meta-analysis comparing adductor canal versus femoral block found adductor canal block preserved the strength of the quadriceps muscle better (p-value = 0.002), although no significant difference in pain control was found [4]. Although regional blocks are emphasized in the literature, specific guidance on managing rebound pain after regional blocks is limited in the reviewed literature. Adjunct systemic analgesics may help mitigate this phenomenon, but further research is needed to establish standardized recommendations.

Long-acting infiltration analgesia around the joint, together with scheduled acetaminophen, non-steroidal anti-inflammatory drugs (NSAIDs) (or cyclooxygenase-2 (COX-2) inhibitors), and gabapentinoids, significantly reduces opioid requirements. Studies have documented that ERAS patients have lower postoperative pain scores and use far fewer opioids than similar patients under traditional care - one systematic review (on both THA and TKA) noted that 87.5% of ERAS reports documented reduced pain and/or opioid consumption relative to conventional protocols [11].

In our institution’s ERAS pathway, we avoid intrathecal morphine and IV patient-controlled opioids, instead favoring modalities that minimize sedation and enable early therapy (e.g., periarticular bupivacaine injections, intravenous dexamethasone for nausea and pain, etc.). The net effect is that patients in the ERAS program typically ambulate sooner and with less discomfort, which further reinforces the recovery cycle.

Prehabilitation and patient optimization

Preoperative “prehabilitation” (exercise and education before surgery) has been proposed as an ERAS component to boost early recovery. A recent systematic review of 22 randomized trials (n = 1,601) found that home-based prehabilitation yielded modest improvements in preoperative pain (e.g., pain score reductions of approximately 0.5-0.7 points on a 10-point scale) and function (e.g., Western Ontario and McMaster Universities Arthritis Index (WOMAC) function improved by 3-5 points on a 0-100 scale) in TKA and THA patients, and significantly reduced hospital LOS for TKA by about 0.4 days (mean difference -0.43 days) [2]. The programs generally prescribed three to five exercise sessions per week, each lasting 20-60 minutes, for a period of four to eight weeks prior to surgery. Programs were predominantly of moderate intensity, with progression guided by patient tolerance or therapist input. Core components included strengthening (quadriceps, hip abductors), flexibility/range of motion, aerobic activity (walking or cycling), and functional training (sit-to-stand, stair climbing). Delivery was via manuals or DVDs, often supplemented by weekly telephone or in-person check-ins to encourage adherence (reported adherence varied). The LOS reduction was statistically significant for TKA (p < 0.001) but not for THA (p=0.12), indicating the benefit was pronounced more in TKA. Pain scores were slightly better at six weeks post-TKA in some trials, but there were no significant differences in longer-term postoperative functional outcomes or quality of life between prehabilitation and control groups.

In summary, prehabilitation tends to improve patients’ pre-op status and may hasten very early recovery (e.g., facilitating discharge approximately 10 hours earlier, which is meaningful in SDD pathways), but evidence is mixed on its effect on longer-term outcomes. Current ERAS guidelines still encourage patient optimization before arthroplasty - including exercise, nutrition, and smoking cessation - as these measures have broad health benefits. However, there is recognition that prehab is not absolutely required for a successful ERAS outcome. Setting realistic expectations and ensuring patients are mobilized as soon as possible after surgery, rather than on any single prehab protocol. Conversely, patient factors like severe frailty or poor health may limit prehab participation; such patients can still benefit from ERAS, though outcomes may not equal those of healthier individuals (this remains an area for further study). Reported barriers to implementing prehab include variable patient adherence, limited access to supervised programs, and costs [2] - issues future research should address.

Blood conservation and surgical technique

Minimizing blood loss is a key ERAS goal to avoid transfusions and speed recovery. Current guidelines strongly endorse tranexamic acid (TXA) for virtually all hip and knee arthroplasties [5]. TXA reduces perioperative bleeding and transfusion requirements without added thromboembolic risk. For example, a recent pilot randomized trial in TKA (40 patients) showed that extending TXA into the postoperative period (oral TXA for three days after surgery, in addition to IV TXA intraoperatively) safely improved early knee flexion and patient-reported function at two to six weeks, with no difference in hemoglobin drop or thrombotic events. The extended-TXA group achieved approximately 10° greater knee flexion at six weeks (116° vs. 106°, p≈0.03) and better pain scores than placebo, without any increase in adverse The authors concluded that prolonged TXA provides better short-term pain and mobility outcomes after TKA [12], although larger trials are needed to confirm long-term benefits. In general, standard TXA dosing (one or two IV doses around the time of surgery) is now routine in ERAS protocols worldwide, given its strong evidence base for reducing blood loss and transfusions [12]. Dosing regimens vary slightly, and specific patient subgroups may need adjustments - for instance, patients with severe renal impairment or a history of thromboembolism may receive lower TXA doses or be evaluated on a case-by-case basis. Notably, studies indicate TXA is safe even in many high-risk patients (no significant increase in VTE with TXA, even in high-risk groups) [12].

ERAS protocols also emphasize meticulous surgical technique to reduce tissue trauma: surgeons often omit routine suction drains (to prevent siphoning off blood and nutrients from the wound) and limit tourniquet use in TKA (releasing it prior to wound closure to restore limb perfusion). These practices have been associated with less pain and swelling postoperatively. For example, a retrospective study noted higher transfusion rates with drains (presumably due to increased blood loss into the drain) and higher LOS [6]. In short, aggressive blood conservation - notably the routine use of TXA and avoidance of unnecessary drains - is now integral to ERAS and clearly contributes to faster recovery and a lower risk of anemia-related fatigue [12].

Hospital LOS

A primary tangible benefit of ERAS in arthroplasty is a reduced hospital stay. Meta-analyses uniformly show shorter LOS with ERAS pathways than with conventional care. For combined elective primary THA and TKA, a large 2024 meta-analysis (47 studies, approximately 77,000 patients) found that ERAS shortened LOS by about 2.6 days on average (weighted mean difference ≈ -2.65 days) [7]. Likewise, another systematic review (approximately 217,000 patients) reported that the vast majority of studies (>95%) observed a significant LOS reduction with ERAS [10]. Importantly, neither of the two major systematic reviews and meta-analyses provides consistent, head-to-head pooled subgroup effect sizes separating total hip arthroplasty (THA) from total knee arthroplasty (TKA). Where subgroup data are reported, both procedures demonstrate clear benefit from ERAS implementation, but the differences in magnitude are small and inconsistent across studies, suggesting that the overall impact of ERAS pathways is broadly comparable between hip and knee arthroplasty [7,10].

In practical terms, these reductions translate to a typical hospital stay of one to three days under ERAS protocols versus three to five days (or more) under traditional care. For example, a recent multicenter randomized trial in TKA showed mean LOS of 5.9 ± 1.2 days under an ERAS pathway versus 8.2 ± 1.8 days under usual care (a >2-day difference, p < 0.001) [13]. The study included American Society of Anesthesiologists (ASA) I-III patients aged 18-80, excluding revisions, bilateral cases, and those with major uncontrolled comorbidities. In older THA patients (elective patients > 60 years with ASA I-III), implementation of an ERAS nursing program also significantly shortened hospitalization (mean ~4 days ERAS vs. ~6 days control) in a 2025 trial [14]. Results were not stratified by discharge destination.

These findings are remarkably consistent across studies: ERAS protocols typically achieve a 30-50% reduction in LOS without compromising patient safety. Patients are discharged sooner and in better functional condition. It’s worth noting that reduced LOS has not led to higher readmission rates, as discussed below, which reassures providers that sending patients home earlier is not causing rebound issues. Moreover, shorter hospital stays bring secondary benefits: lower exposure to hospital-acquired infections, more rapid return to a familiar home environment (which many patients prefer, and may help impede delirium), and reduced cost per case. Indeed, health economic analyses show that ERAS programs substantially cut costs primarily by shaving inpatient days. One Australian budget-impact study projected that expanding ERAS nationwide could save over AUD 600 million in hospitalization costs from 2023 to 2030 (a modelled projection of a return on investment (ROI) around 8.9) [15].

It is worth noting that unplanned healthcare contacts, such as emergency department visits or primary care consultations, were not systematically evaluated, leaving this aspect underexplored. Additionally, studies included in the meta-analyses were conducted predominantly in high-income countries, most commonly Europe (United Kingdom, Scandinavia, Germany, and the Netherlands), North America (U.S. and Canada), and parts of Asia (notably China and South Korea). Representation from low- and middle-income countries was minimal, and no included trials originated from sub-Saharan Africa or South America. Accordingly, while the pooled findings strongly support ERAS efficacy in elective THA and TKA, their generalizability to lower-resource settings may be limited.

**Figure 1:**
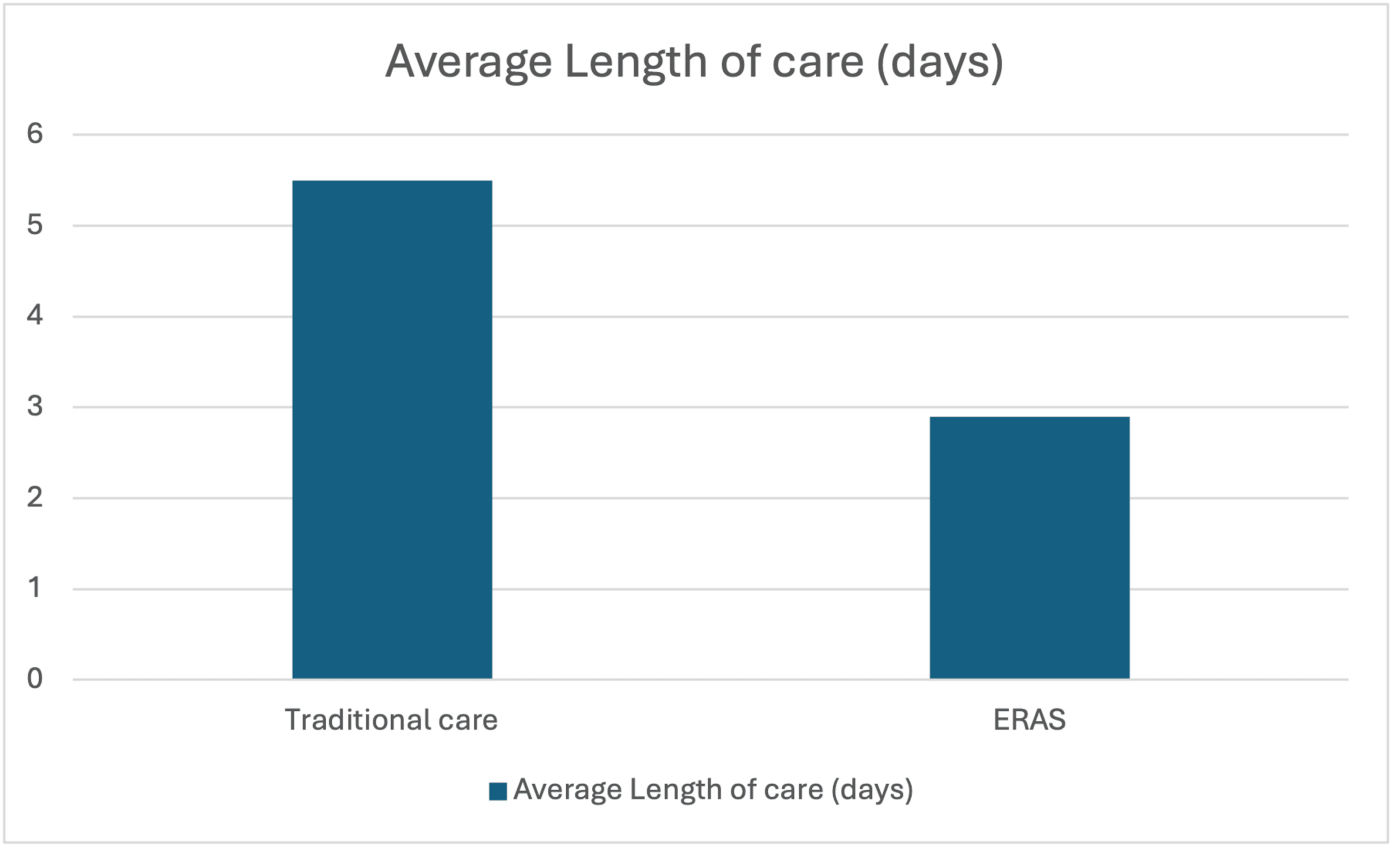
Average hospital length of stay (LOS) for joint replacement: ERAS pathway vs. traditional care. ERAS protocols dramatically shorten LOS without increasing readmissions. ERAS: enhanced recovery after surgery Source: Created using data from reference [7].

The bar chart (Figure 1) below compares average postoperative LOS for TJA under conventional protocols versus ERAS protocols. Enhanced recovery patients are often discharged in approximately one to three days (or even same-day for qualifying cases), whereas historically patients stayed nearly a week. This >50% reduction in LOS is supported by large meta-analyses [7] and reflects the impact of early mobilization and streamlined care.

Complications and readmission rates

Meta-analytic data show no statistically significant difference in overall postoperative complications or in 30-day readmissions under ERAS care compared to traditional protocols [16]. In fact, some studies report modest reductions in complications with ERAS. For example, the randomized trial of ERAS versus standard care in older THA patients (60 years) found a lower incidence of postoperative problems such as delirium, urinary retention, and wound problems in the ERAS group [14]. In that and other trials, complications were generally assessed using routine clinical documentation rather than blinded adjudication, which may underestimate milder events but captures those requiring medical attention. The large multicenter POWER2 study from Spain reported no difference in overall complication rates between ERAS and non-ERAS groups (about 10% in both, p = 0.22), but did find significantly fewer moderate-to-severe complications in the ERAS group - defined as events requiring substantial medical or surgical intervention, corresponding approximately to Clavien-Dindo grade ≥III (4.6% vs. 6.1%, odds ratio (OR) 0.74, p = 0.02) [17]. Moreover, hospitals with the highest adherence to ERAS elements had the lowest complication rates, suggesting that protocol fidelity influences outcomes. Importantly, the POWER2 follow-up was restricted to 30 days, so it remains uncertain whether these benefits persist longer term.

Notably, none of the large trials or meta-analyses have detected higher 30-day readmission rates with ERAS. An earlier meta-analysis even found that 30-day postoperative mortality was significantly lower in ERAS patients (0.3%) compared to controls (0.7%) - roughly a 50% relative risk reduction [18]. However, the absolute difference was only ~0.4%, so the clinical impact is small, and the mortality effect was not always consistent after adjusting for confounding variables. This finding, therefore, supports the safety of ERAS but should not be overstated.

Taken together, the weight of evidence indicates that ERAS safely compresses the recovery timeline: patients go home sooner without paying a price in terms of higher complication or readmission rates. The overall trend is toward equal or better clinical outcomes, particularly when protocols are consistently implemented. This speaks to the success of the multimodal, team-based ERAS approach in controlling the factors that typically cause complications (immobility, opioids, fluid imbalance, etc.). Nevertheless, because most data come from high-volume centers with mature ERAS programs, the impact in lower-volume hospitals or during initial implementation may be less dramatic - highlighting the importance of institutional readiness, effective training, and early outcome tracking.

Pain control and opioid consumption

ERAS pathways place a strong emphasis on opioid-sparing analgesia. The multimodal approach - combining regional blocks, local anesthetic infiltration, non-opioid analgesics, and judicious short-term opioid use - has translated into better pain control and markedly lower opioid use in most studies. As noted above, a recent review found that in seven of eight reports (87.5%), ERAS patients had reduced postoperative pain scores, opioid requirements, or both compared with those receiving conventional perioperative care [11]. In the cited randomized trials, pain was measured using standardized scales such as the visual analogue scale (VAS) and numeric rating scale (NRS), with moderate-to-severe pain typically defined as VAS/NRS > 4/10. For instance, the Chinese multicenter randomized trial in TKA demonstrated that on postoperative day 1 (POD1), significantly fewer ERAS patients reported moderate-to-severe pain at rest and with movement compared to controls [13]. Likewise, the RCT in older THA patients (>60 years) showed that ERAS nursing care led to lower pain scores in the first 48 hours after surgery, while using less morphine than the standard-care group [14]. Non-opioid pharmacologic regimens in these studies included acetaminophen, NSAIDs (e.g., celecoxib or ibuprofen), and occasionally gabapentinoids or COX-2 inhibitors. By contrast, traditional perioperative models often relied on general anesthesia and higher doses of systemic opioids, leading to more sedation, nausea, and delirium that impeded recovery.

In our clinical experience, ERAS patients frequently ambulate within hours of surgery with minimal pain, something that was rare in the opioid-heavy model of the past. Quantitatively, many centers have reported dramatic decreases in opioid consumption under ERAS. For example, one hospital noted a reduction of approximately 72% in median inpatient opioid use after implementing ERAS, and up to 15-20% of their arthroplasty patients required no opioids at all postoperatively. Importantly, these opioid reductions have not compromised pain control; ERAS patients often reported equal or better pain relief while using far fewer narcotics. This opioid-sparing success helps avoid side effects (nausea, constipation, respiratory depression) and has implications for the opioid epidemic - patients who start with fewer opioids are less likely to become chronic users. Long-term opioid outcomes (beyond discharge) were included in only a few reports - with the TKA cohort showing sustained opioid avoidance at three months - but broader data on persistent or chronic use remain limited. Similar to other outcomes, it should be noted that many of these studies were conducted in specific healthcare settings (high-volume specialized centers). Their direct applicability to broader populations warrants cautious interpretation.

Overall, ERAS protocols demonstrate that optimal pain management is achievable with minimal reliance on opioids, through a combination of regional anesthesia, non-opioid pharmacologics, and early mobilization to reduce stiffness.

Functional recovery and patient-reported outcomes

By design, ERAS seeks to accelerate functional recovery after joint replacement. Multiple studies show that ERAS patients ambulate earlier and regain strength faster than those under traditional protocols. For example, a recent randomized trial in THA compared an ERAS regimen to conventional care and found significantly better early mobility in the ERAS group: ERAS patients had faster Timed Up-and-Go test results and could walk longer distances by PODs 2-3 (p < 0.05). Ambulation distance was recorded by physiotherapy staff on the ward, and “independence” in the floor- and chair-rise tasks was defined as completing the movement without hands-on assistance. They also achieved independence in floor and chair rise tasks sooner. These functional gains occurred without any increase in pain or decrease in satisfaction [19]. Patient satisfaction in that trial was assessed using the PPP33 (patient assessment in the perioperative phase) instrument and was consistently high in the ERAS arm; results were not stratified by surgical approach or surgeon volume, so independence of satisfaction from technique/volume cannot be fully determined [19]. Patients appreciate the rapid recovery and often express that they would undergo the same protocol again. The follow-up period in this RCT was limited to one week; therefore, generalizability to patients with higher frailty/comorbidity burdens or different cultural/health-system contexts remains uncertain, and mid-/long-term functional trajectories were not captured.

**Figure 2:**
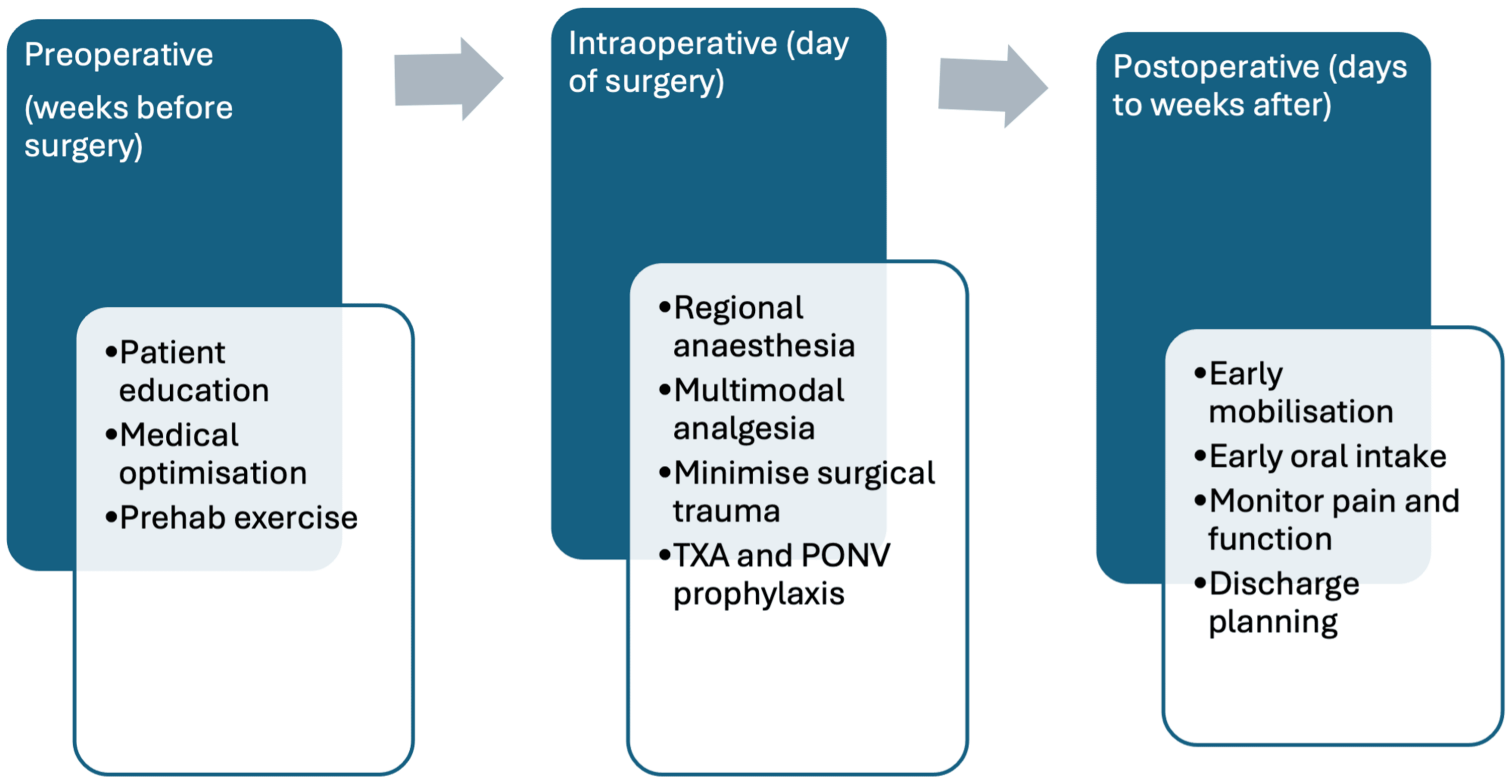
Timeline of ERAS interventions in total joint arthroplasty, spanning preoperative, intraoperative, and postoperative phases. Each phase includes targeted strategies that collectively enhance recovery. TXA: tranexamic acid; PONV: postoperative nausea and vomiting Source: Created using data from references [1,7,8,11].

Most studies that track PROMs have found no detrimental effect of ERAS on medium-term outcomes. At six weeks to three months post-op - when improvements from joint replacement become evident - ERAS patients tend to have similar scores on standardized metrics (such as the Oxford Hip/Knee Score, WOMAC, or Knee Injury and Osteoarthritis Outcome Score (KOOS)) as patients who underwent traditional pathways. Where differences are seen early, they most commonly appear in pain and quality-of-life domains (e.g., pain subscales, general health) rather than large, durable changes in composite function. By 6-12 months after surgery, virtually all patients have comparable outcomes regardless of pathway, reflecting that the implant and rehabilitation ultimately drive long-term success more than the hospital stay does. Crucially, ERAS does not trade speed for efficacy: the magnitude of pain relief and functional improvement from pre-op to one-year post-op is at least as good under ERAS as with conventional care. For example, one hospital’s fast-track (single-center) program reported that 93% of hip replacement patients and 86% of knee replacement patients were satisfied with their surgical outcome at one year (and >95% would undergo the surgery again), outcomes on par with or better than historical norms [20]. High long-term implant survivorship has also been reported under ERAS programs (in one series, two-year revision rates under 1% [20], which is within expected ranges, but registry-level confirmation would strengthen robustness). In summary, ERAS accelerates the early recovery phase - getting patients on their feet and functional days sooner - without compromising the ultimate gains in pain relief, mobility, and quality of life that joint arthroplasty offers. Figure 2 shows the timeline of ERAS interventions in TJA from the preoperative to postoperative phases.

SDD (outpatient) arthroplasty 

ERAS protocols have paved the way for a major expansion of outpatient (same-day) hip and knee arthroplasty. Outpatient TJA is defined as discharge on the day of surgery, typically within approximately 8-12 hours post-op (discharge timing may vary by region and protocol). Recent series report high success and safety in this setting for carefully selected patients. Published success rates for SDD range from approximately 70% up to >90% in eligible cohorts [21]. These percentages generally reflect “successful discharges,” meaning patients discharged home as planned; failed discharges (unplanned overnight stays due to pain, nausea, urinary retention, or logistical factors) were counted in the denominator, so true success is somewhat lower than the headline figures.

For example, in the United Kingdom, a day-case program implemented at an NHS hospital achieved SDD in 47% of THA and 67% of unicompartmental knee arthroplasty (UKA) cases in its first year [22]. Another report from Canada noted an 83% SDD rate among hip and knee patients enrolled in an ERAS “FAST track” pathway [23]. In the U.S., ASCs routinely achieve >90% outpatient discharge rates for primary joint replacements - one matched-cohort study at a Tennessee ASC had essentially 100% of patients (in both the spinal and general anesthesia groups) discharged home same-day [3]. However, that ASC cohort included predominantly privately insured patients, which may limit generalizability to more diverse insurance populations.

It is important to emphasize that patient selection is key for outpatient arthroplasty. Typical selection criteria include age under ~75, relatively healthy (e.g., ASA I-III with well-controlled comorbidities), BMI not extreme (commonly < 35 kg/m2) [24], motivated patient with good social support, and surgery scheduled early in the day. Caregiver readiness and home environment were variably assessed: in some programs, this was formally evaluated by nursing staff or physiotherapists, while in others it was self-reported by patients and families. When these conditions are met, very high outpatient success is possible. Notably, partial knee replacements (UKA) tend to have the most favourable conditions with SDD due to less surgical stress - indeed, a systematic review found an overall 88% successful SDD rate after UKA [25]. Table 3 summarizes the typical criteria for outpatient TJA; these vary by region and health-system policy rather than being universally standardized.

**Table 3:** Typical criteria for same-day discharge (outpatient) total joint arthroplasty. ASA: American Society of Anesthesiologists; BMI: body mass index; TJA: total joint arthroplasty; MI: myocardial infection; COPD: chronic obstructive pulmonary disease; ERAS: enhanced recovery after surgery Source: Created using data from references [24,9,26,27].

Factor	Criteria for Outpatient (Same-Day) Discharge
Age	Generally < 75-80 years old [24]. (Younger patients tend to tolerate rapid recovery better. Very elderly patients are approached cautiously for same-day discharge.)
ASA status	Prefer ASA I-II (healthy or mild systemic disease). ASA III can be considered if comorbidities are well-controlled. Avoid ASA IV (severe disease that is a constant threat to life) for outpatient TJA [26].
BMI	BMI in a moderate range (commonly < 35 kg/m^2^) [24]. Higher BMI increases risk of complications (wound issues, OSA) and may preclude same-day discharge in many programs.
Comorbidity control	Well-controlled medical conditions. No significant cardiac disease (e.g., recent MI or unstable angina, uncontrolled arrhythmia). No uncontrolled diabetes (avoid outpatient TJA if insulin-dependent with poor control) [26]. No untreated obstructive sleep apnea requiring overnight monitoring. No active bleeding disorders, cirrhosis, end-stage renal disease, or other major unstable conditions [26]. (Any chronic conditions such as hypertension, COPD, etc., should be optimized pre-op.)
Home support	Responsible adult at home on the day of surgery and the first night. Patient must have a caregiver or family member to assist with transportation, basic needs, and monitoring for at least 24-48 hours post-op [27]. The home environment should be safe (e.g., fall hazards addressed).
Patient motivation and compliance	Highly motivated, well-educated patient. The patient should be engaged in the decision for outpatient TJA, understand the ERAS pathway, and be compliant with instructions (e.g., doing prehab exercises, adhering to post-op rehabilitation) [9]. Setting appropriate expectations through thorough preoperative education is crucial - patients should feel comfortable with early discharge and confident in managing recovery at home.

Comparative studies consistently show similar or even lower complication and readmission rates for outpatient arthroplasty versus inpatient stays. For instance, the UK day-case series reported no increase in 30-day complications or reoperations compared to traditional inpatients, and the early functional outcomes and patient-reported scores were equivalent between the groups [22]. In the U.S., a study focusing on Medicare-age patients found that outpatient TKA can be performed safely even in this older cohort, with no difference in 30-day readmissions compared to those kept overnight [28]. Similarly, the ASC study by Calkins et al. noted no significant differences in 90-day adverse events or readmissions between their outpatient spinal versus outpatient general anesthesia groups [3]. While readmission rates were not higher, a few studies did note increased unscheduled telephone contacts or outpatient service calls in SDD groups, suggesting that closer follow-up is needed to manage minor concerns.

On a system level, national database analyses have observed that as ERAS and outpatient practices became more widespread, the overall complication burden after TJA has actually decreased over the past decade - likely a reflection of better protocols and patient optimization [26]. Notably, a “day-case effect” has been observed: instituting a formal SDD pathway can reduce the LOS for all arthroplasty patients in a unit, even those who ultimately stay overnight. In the UK series, after the day-case protocol was introduced, the mean LOS fell by 0.7 days for THA, 0.6 days for UKA, and 0.5 days for TKA across the board (p < 0.001) [22]. This effect was sustained beyond the initial implementation period, with reductions persisting into subsequent years as the pathway matured, though the largest drop occurred during the first year of rollout. Thus, ERAS-driven innovations tend to elevate the efficiency of care for all patients, not just the healthiest.

From the patient’s perspective, satisfaction and early functional recovery remain high under outpatient ERAS pathways. Many patients report that recovering in the comfort of their home is preferable as long as pain is well-controlled. Pain control after discharge typically relied on scheduled acetaminophen plus NSAIDs, with rescue short-course opioids available if needed; structured written instructions for rescue analgesia were common. Studies show that patients discharged the same day have pain relief and mobility improvements comparable to those of inpatients on PODs 1-2. In fact, some reports suggest early PROMs may be better: one fast-track hip study found that 91% of ERAS patients felt “much better” or “better” in the early postoperative period than before surgery (versus 78% in the conventional-care group), a difference that reached statistical significance. Importantly, long-term PROMs (6-12 months) are not worse - and in some cohorts even slightly improved - for ERAS/outpatient patients [22].

SDD has generally been more successful after UKA and THA than TKA, reflecting differences in surgical invasiveness; outpatient TKA is feasible but has somewhat lower success rates and a higher likelihood of failed discharges due to pain or stiffness. Key strategies to achieve safe SDD include scheduling surgery early, avoiding long-acting intrathecal opioids (which can delay mobilization due to side effects), coordinating physical therapy on the day of surgery, and ensuring thorough patient and caregiver education with close postoperative follow-up.

International perspectives on ERAS

ERAS adoption in TJA has grown worldwide, though uptake varies with healthcare systems and local practices. In the United Kingdom, ERAS pathways (often as part of the NHS “Getting It Right First Time” initiative) have significantly shortened LOS and expanded day-case arthroplasty. The UK example cited above - 47% of hip replacements and 67% of partial knees were discharged the same day in an NHS hospital - demonstrates feasibility in a public healthcare setting. Interestingly, introducing that day-case protocol appeared to “pull” average LOS down for even standard patients by about half a day. In Canada, similar successes are reported: a quality-improvement project (18 months of baseline data ending October 2023) at a community hospital achieved an 83% SDD rate among eligible hip and knee patients by using ERAS and strict selection criteria [23]. These programs have shown that with proper patient prep and post-discharge support (e.g., home physiotherapy and nurse check-ins), many arthroplasty patients do not require an inpatient stay.

In the U.S., the drive toward outpatient TJA has been accelerated by healthcare economics and patient preference. Between 2012 and 2020, the proportion of TKA cases performed on an outpatient basis in the U.S. increased from only 0.4% to 14.1%, a dramatic rise attributable in part to Medicare policy changes removing TKA from the inpatient-only list [29]. High-volume centers and specialized orthopedic hospitals have embraced ERAS to facilitate this transition. Recent U.S. studies mirror the UK and Canadian findings: with ERAS, both spinal and general anesthesia approaches can yield >98% SDD success in ASCs, and hospital-based programs report outpatient success rates well above 70-80% in selected patients. Importantly, payers in the U.S. are now incentivizing shorter stays (through bundled payments), which aligns with ERAS principles.

Elsewhere, many European nations have robust ERAS programs. The Scandinavian countries (Denmark, Sweden) were early pioneers of “fast-track” arthroplasty and routinely achieve average LOS less than two days for THA/TKA. Denmark, in particular, has leveraged ERAS so effectively that by 2019, over 20% of all joint replacements nationally were outpatient (SDD), up from 6% in 2013 [30]. This was accomplished by systematically optimizing perioperative care across hospitals - essentially standardizing ERAS protocols nationally - such that even before formal outpatient programs, most patients only stayed one night.

In Australia, ERAS is gaining momentum with an eye on cost savings for the healthcare system. As mentioned, a recent Australian analysis projected enormous economic benefits to widespread ERAS implementation. One study from a Melbourne hospital found that switching appropriate cases to a two-day ERAS pathway (from the traditional ~4-day stay) cut direct costs per patient by about 30-50%. In Denmark, a detailed time-driven activity-based costing study similarly showed that outpatient joint arthroplasty can be roughly 40-50% less costly than inpatient surgery, when factoring in ward staffing and facility utilization [31]. Both analyses were limited to short-term direct costs and did not account for long-term outcomes such as revision, chronic pain, or sustained functional gains.

However, some fee-for-service healthcare systems may face inertia or disincentives - for instance, in certain regions, surgeons or hospitals might be financially rewarded for longer stays or higher utilization of inpatient services, which can slow ERAS adoption. Additionally, resource limitations (such as lack of outpatient rehab services or home nursing support) and clinician resistance to change can pose barriers in various locales, including some low- and middle-income countries. Nonetheless, the global trend is toward embracing ERAS. The pace of adoption is uneven, but knowledge transfer through international ERAS society guidelines and collaborative initiatives is helping disseminate best practices.

Finally, ERAS protocols may need tailoring to local contexts. Differences in anesthesia practice (e.g., some countries use more general anesthesia vs. regional) or cultural expectations (e.g., patients’ desire for longer convalescence vs. quick discharge) can influence how ERAS is implemented. Some regions may emphasize certain ERAS elements over others due to resource availability - for example, a hospital without easy access to nerve block expertise might rely more on oral analgesics and other modalities. Despite these variations, the core principles of ERAS (minimizing stress, optimizing pain control and mobility, avoiding unnecessary delays) remain universally applicable. Ongoing international research - including multi-center ERAS audits and registries - will help identify how ERAS can best be adapted to different healthcare environments, including those with limited resources. Table 4 compares the outcomes between ERAS and traditional care.

**Table 4:** Comparison of outcomes between ERAS and traditional care in TJA ERAS: enhanced recovery after surgery; THA: total hip arthroplasty; TKA: total knee arthroplasty; PCA: patient-controlled analgesia; DVT: deep vein thrombosis; TJA: total joint arthroplasty; VAS: Visual Analogue Scale Source: Created using data from references [7,5,11,13,14,8,9].

Outcome	Traditional Care	ERAS Pathway
Length of stay (LOS)	Prolonged hospital stay (historically ~5-7 days for THA/TKA)	Significantly shorter LOS. Meta-analyses show ERAS reduces LOS by ~2-3 days on average [7]. Many patients meet discharge criteria in ~1-3 days, and same-day discharge is often feasible for selected cases.
Pain (acute postop)	Higher pain scores with reliance on opioids for relief.	Lower postoperative pain scores. ERAS cohorts report reduced pain in early recovery - e.g., ~0.9-point lower VAS pain on average with multimodal analgesia [7] - while achieving equal or better analgesia through non-opioid modalities.
Opioid consumption	High opioid use is common (traditional PCA and routine opioids).	Dramatically reduced opioid use. ERAS protocols cut postoperative opioid requirements by >50% in many studies [5,11,13,14]. Patients receive scheduled non-opioids and regional anesthesia, resulting in fewer opioid side effects and earlier mobilization.
Complication rates	Baseline complication incidence (risks of DVT, infection, etc., under standard care).	No increase in complications; often reduced. ERAS does not raise overall complication rates [3]. Some large studies even show significantly fewer complications in ERAS vs. control groups (likely due to reduced bed rest, better pain control, etc.) [8].
Readmission rates	Typical 30-day readmission rates (~3-5% in TJA) under standard care.	Comparable readmission rates. Fast-track programs achieve similar 30-day readmission rates to traditional protocols [11]. Careful patient selection and early follow-up help prevent increased readmissions in ERAS cohorts.
Patient satisfaction	Generally high (most patients are satisfied with surgery, but a longer hospital stay can be a dissatisfier).	Very high satisfaction with ERAS. Patients appreciate faster recovery and discharge. Surveys show that >90% of ERAS patients would choose the same accelerated recovery pathway again [9]. Overall satisfaction scores are significantly higher in ERAS groups, provided expectations are well-managed.

## Conclusions

ERAS pathways in TJA represent a paradigm shift in perioperative management from a traditional, clinician-centered model to a patient-centered, evidence-driven approach that prioritizes rapid recovery without compromising safety. Across numerous studies and diverse healthcare systems, ERAS has demonstrated the capacity to shorten hospital stays (typically by one to three days), substantially reduce complication rates (particularly moderate-to-severe complications), and lower opioid requirements, while concurrently maintaining or enhancing patient satisfaction and outcomes. These improvements, achieved through multidisciplinary coordination and adherence to best practices, have established a new standard of care in hip and knee replacement surgery. Nevertheless, outcomes are not uniform across all settings; there is heterogeneity in adoption and patient selection. Most published successes originate from high-volume centers or integrated health systems with dedicated ERAS teams, whereas smaller or resource-limited hospitals may encounter barriers to implementation, and the benefits may be attenuated during early implementation until protocols mature. It is also necessary to temper optimism with acknowledgment of persistent evidence gaps: for instance, long-term functional outcomes beyond one year under ERAS remain underexplored, and optimal strategies for specific patient subgroups (such as those with severe comorbidities or frailty) remain under investigation. Nonetheless, the evidence to date indicates that ERAS confers dual advantages for patients and healthcare systems, simultaneously improving clinical outcomes and efficiency.

In future directions, efforts should focus on broader implementation and pathway optimization. Key priorities include enhancing clinician education and engagement (to mitigate implementation resistance), ensuring adequate infrastructure to support outpatient recovery (including home physiotherapy and telehealth follow-up), and tailoring ERAS elements for special populations such as elderly, obese, or medically complex patients. Sustaining high compliance with ERAS protocols in real-world practice settings is also critical. Future research should address unresolved research questions, including identifying which ERAS components yield the greatest marginal benefit (to further optimize protocols) and evaluating patient-reported outcomes and quality of life across extended postoperative trajectories. Finally, as ERAS becomes increasingly adopted worldwide, collaborative networks and registries will play a pivotal role in continuous quality improvement, enabling centers to share data, audit outcomes, and collectively advance the standard for perioperative care.
